# Recent insights into the role of microbiome in the pathogenesis of obesity

**DOI:** 10.1177/17562848221115320

**Published:** 2022-08-09

**Authors:** Eduard W. J. van der Vossen, Marcus C. de Goffau, Evgeni Levin, Max Nieuwdorp

**Affiliations:** Department of Experimental Vascular Medicine, Amsterdam University Medical Center, University of Amsterdam, Amsterdam, The Netherlands; Department of Experimental Vascular Medicine, Amsterdam University Medical Center, University of Amsterdam, Amsterdam, The Netherlands; Department of Experimental Vascular Medicine, Amsterdam University Medical Center, University of Amsterdam, Amsterdam, The Netherlands; Horaizon BV, Delft, The Netherlands; Department of Internal and Vascular Medicine, Amsterdam University Medical Center, Meibergdreef 9, room D3-211, Amsterdam, 1105 AZ, The Netherlands; Department of Experimental Vascular Medicine, Amsterdam University Medical Center, University of Amsterdam, Amsterdam, The Netherlands

**Keywords:** guilds, gut microbiome, gut microbiota, FMT, machine learning, obesity, trophic networks

## Abstract

Obesity is a risk factor for many chronic diseases and its rising prevalence the last couple of decades is a healthcare concern in many countries. Obesity is a multifactorial problem that is not only limited in its causation by diet and lack of exercise. Genetics but also environmental factors such as the gut microbiome should similarly be taken into account. A plethora of articles have been published, that from various different angles, attempt to disentangle the complex interaction between gut microbiota and obesity. Examples range from the effect of the gut microbiota on the host immune system to the pathophysiological pathways in which microbial-derived metabolites affect obesity. Various discordant gut microbiota findings are a result of this complexity. In this review, in addition to summarizing the classical role of the gut microbiome in the pathogenesis of obesity, we attempt to view both the healthy and obesogenic effects of the gut microbiota as a consequence of the presence or absence of collective guilds/trophic networks. Lastly, we propose avenues and strategies for the future of gut microbiome research concerning obesity.

## Introduction

The continuous and rapid increase in the prevalence of obesity the last couple of decades has been a major healthcare concern in many countries, particularly now in the era of coronavirus disease 2019. Obesity is a risk factor for an expanding set of chronic diseases including cardiovascular disease,^1,2^ type 1 diabetes and type 2 diabetes (T2D),^1^ chronic kidney disease,^1^ non-alcoholic fatty liver disease,^3^ many cancers^1,4^ and arthritis in weight-bearing joints.^5,6^ Obesity, as defined by the excessive accumulation of body fat to such an extent that health may be adversely affected, is frequently assessed using the body mass index (BMI; weight in kilograms divided by the square of the height in meters). Obesity is a multifactorial problem which is not just limited in causation by diet or a lack of exercise but one which also includes genetic, environmental and psychosocial factors that act through physiological mediators of energy intake and expenditure.^7^ The gut microbiome is one of these environmental factors; the link between fat storage and the gut microbiome has been established in mice studies almost two decades ago.^8^ Faecal microbiota transplantation (FMT) studies provide an even more tangible layer of proof.^9,10^ By now a plethora of studies have been performed investigating the gut microbiome and factors associated with it in relation to obesity. These include association and mechanistic studies tackling the roles of the individual gut microbes involved in obesogenic physiology, ones that mainly focus on immunological factors and others that attempt to link microbes to host metabolism and the innate immune system.^11^ It is crucial though to keep in mind that many associations and correlations published in this field (or elsewhere) should not be mistaken for proof of causation. Many intervention studies that do provide evidence of causation have furthermore been performed in mice and may not always directly apply to humans. This review covers the different angles, both by looking at the role(s) of single bacteria but especially by placing an emphasis on the microbiome composition as a whole, looking at guilds and trophic networks of bacteria/archaea in an effort to disentangle the complex relation of the gut microbiome and obesity.

## Modalities by which the gut microbiome affects the pathogenesis of obesity

### Immunological responses related to obesity

**Figure 1.**
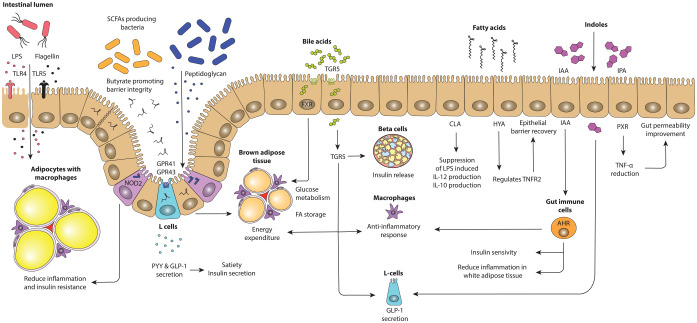
Putative mechanisms by which the gut microbiota can influence host metabolism. Parts of the gut microbiota, such as flagellin, and LPS bind to TLRs,^24,27^ whereas intracellular NOD2 senses peptidoglycan.^28^ Production of several SCFAs can bind to GPR41 and GPR43 leading to increased expression of PYY and GLP-1.^29^ Bile acids activate TGR5 and FXR affecting lipid and glucose metabolism.^30,31^. Fatty acids, such as HYA, regulate TNFR2, involved in epithelial barrier recovery.^32^ Indoles influence the host metabolism *via* GLP-1 modulation^33^ and activation of AHR and binding to PXR.^34,35^ AHR, aryl hydrocarbon receptor; GLP-1, glucagon-like peptide-1; GPR, G protein-coupled receptor; HYA, 10-hydroxy-cis-12-octadecenoic acid; IAA, indole-3-acetic-acid; IPA, indole-3-propionic acid; LPS, lipopolysaccharide; NOD2, nucleotide-binding oligomerization domain 2; PXR, pregnane X receptor; PYY, peptide YY; SCFAs, short-chain fatty acids; TGR5, Takeda G protein-coupled receptor; TLRs, toll-like receptors; TNFR2, tumour necrosis factor receptor 2.

An association between the gut microbiota and the host immune system has been established by many researches.^8,11–18^ A frequently recurring finding is that obesity is related to microbially induced chronic low-grade inflammation.^16,17,19,20^ The close contact between the microbiota and intestinal cells is mediated by microbial-associated molecular patterns that can bind to pattern recognition receptors (PRRs) in the epithelial and immune cells.^21^ These PRRs belong to the innate immune system and control inflammatory and immunological responses. PRRs can also detect damage-associated molecular patterns released from host cells.^14^ As an example, Cani *et al.*^11^ in 2007 showed that lipopolysaccharide (LPS), a component of the outer membrane of gram-negative bacteria, appears to cause low-grade inflammation in mice. A similar observation was made in a human study in which energy intake was associated with endotoxemia and concomitantly inflammation.^22^ Indeed, in subjects with T2D, gram-negative bacteria, including Proteobacteria and Fusobacteria, were significantly more abundant compared to healthy controls.^23^ LPS causes inflammation *via* the LPS receptor cluster of differentiation 14 (CD14) and co-receptor toll-like receptor (TLR)4,^24^ which, in turn, leads to increased production of proinflammatory cytokines by adipocytes. Interestingly, the type of diet plays an important role. Pectins were shown to inhibit LPS-induced TLR4 activation in monocytes or dendritic cells,^25^ whereas a fructose or high-fat diet led to an increase in LPS-containing Proteobacteria causing TLR4-mediated inflammation in the liver^24,26^ (Figure 1) Leptin signalling, which is involved in satiety and perturbed energy balance,^15^ is consequently dysregulated. It has also been shown that secreted lipoprotein lipase (LPL) inhibitor angiopoietin-like protein 4 (a fasting-induced adipose factor) can be suppressed by the microbiota, which, in turn, leads to increased LPL activity and fat storage in white adipose tissue.^8^

Another example is peptidoglycan, a component of the bacterial cell wall, which is of importance for homeostasis.^17^ Nucleotide-binding oligomerization domain 2 (NOD2), a cytosolic PRR positioned inside epithelial cells and immune cells, is able to sense muramyl dipeptide which is a small product of peptidoglycan.^28^ This PRR is crucial for immune response during pathogen invasion and several inflammatory disorders and thus regulating mucosal bacterial colonization.^21^ NOD2-negative mice shown to have increased adipose tissue, liver inflammation and insulin resistance during high-fat diet^17^ and are hence often used in diabetes studies. In obese mice with functioning NOD2 receptors, muramyl dipeptide recognition shown to have reduced adipose inflammation and insulin resistance without weight loss or altering the gut microbiota composition.^36^

The gut microbiota is also linked to the immune system *via* TLR5, which are positioned on epithelial cells. Compared to the wild-type germ-free mice, TLR5-deficient mice have increased levels of insulin resistance and adiposity. Gut microbiota transfer from these TLR5-deficient mice to wild-type germ-free mice also led to the transfer of similar features of metabolic syndrome in these wild-type mice.^16^ The immune system senses the gut microbiota composition and localization of the intestinal microbiota *via* TLR5 to avoid dissemination of commensal gut microbiota to extraintestinal organs, overgrowth of toxigenic members, and overgrowth and invasion of opportunistic pathogens.^37^ Flagellin detection by TLR5 causes the production of IL22, thereby preventing diseases associated with intestinal inflammation.^27^ A study investigating mice that lack TLR5 receptors observed a loss of flagellin-specific immunoglobulins leading to an increase in flagellated bacteria, including many Proteobacteria, and increased mucosal barrier breakdown and inflammation.^18^ Indeed, obese humans tend to have higher amounts of faecal flagellin, reduced amounts of faecal anti-flagellin IgA and higher levels of chronic intestinal inflammation compared to lean subjects.^38^

### The role of short-chain fatty acids

Short-chain fatty acids (SCFAs) are important microbially derived end products of microbial anaerobic fermentation that have a multitude of effects on the host. It is a group of carboxylic acids with fewer than six carbons, including acetate, propionate and butyrate. Some of the acetate is consumed to produce butyrate^39,40^ but the typical colonic ratio of acetate to propionate to butyrate is 3:1:1, respectively. These SCFAs have a multitude of (beneficial) effects in several different tissues (exquisitely reviewed by den Besten *et al.*^29^). SCFAs are considered energy sources and energy regulators for the human host, but they can also contribute to maintaining homeostasis of the intestinal environment. Extracellular activity of SCFAs is among others mediated by the G protein-coupled receptors (GPRs), also known as free fatty acid receptor 2 (FFAR2; GPR43) and FFAR3 (GPR41). These receptors are expressed on a wide range of cells, including gut epithelial cells, adipocytes, enteroendocrine L cells, innate immune cells and neurons of the somatic sensory ganglia.^41,42^ *Via* such mechanisms, SCFAs are involved in the regulation of the peptide YY and glucagon-like peptide 1 (GLP1) hormones, produced by L-cells. Both of these hormones regulate satiety in the nervous system and GLP1 also plays a role in glucose-stimulated insulin sensitivity and secretion.^43–45^ Satiety is also controlled by propionate *via* activation of FFAR3 in adipocytes, as these adipocytes produce leptin.^46^ Both microbially derived butyrate and propionate induce intestinal gluconeogenesis, which, in turn, induces beneficial effects on glucose and energy homeostasis.^47^ It has furthermore been shown that butyrate stimulates the activation of brown adipose tissue *via* the activation of FFAR2, substantially contributing to energy expenditure^45,48^ and that fat accumulation is suppressed by butyrate-induced FFAR2 activation in white adipose tissue.^49^ Finally, butyrate has been shown to reduce bacterial translocation in epithelia by decreasing the permeability of the intestinal barrier.^50–52^

Within the gut, the production of SCFAs occurs *via* various intermediates.^53^ Various species, utilizing different enzymes for each of the steps leading to these intermediates and/or end products, are involved in this process.^54,55^ A multitude of options for each of these steps or for alternative routes exists within a microbial community and it hence depends on the microbial composition what the SCFA fermentation profile looks like and how this profile is achieved. These different routes, or chains of conversion, can be considered the basis of various trophic networks of microbial species where various species benefit from the presence of other microbial species *via* syntrophy (cross-feeding). An example of cross-feeding is the utilization of carbohydrates by *Bifidobacterium adolescentis* resulting in the production of acetate. Acetate can subsequently be further utilized by *Faecalibacterium prausnitzii* using the butyryl-CoA:acetate CoA-transferase pathway to produce butyrate.^55^ *Anaerobutyricum hallii* and *Anaerostipes caccae* produce butyrate using a different metabolic pathway consuming both the lactate and the acetate produced by *B. adolescentis*.^40,56^ A (severe) disturbance of the microbial profile (dysbiosis) in critically ill children, as occurs during (chronic) disease and/or antibiotic use, has been shown to be detrimental in terms of the fermentative capability of the gut microbiome resulting in extremely reduced production levels of butyrate, propionate and acetate (and increased levels of intermediates such as lactate) which is not conducive for their recovery and might cause additional comorbidities.^57^ In T2D, a common trend seen in many studies is that the abundances of butyrate producers, such as *Roseburia* and *Faecalibacterium*, are lower in diabetics than in controls.^58–63^ The opposite, dependant on diet, might however also be true in obesity. Germfree mice were protected against the obesogenic and inflammatory effects induced by eating a Western-style, high-fat, sugar-rich diet. An overproduction of SCFAs might lead to a higher energy availability and intake.^64^ Indeed, a study comparing obese to lean subjects showed that obese individuals had higher total SCFAs levels, though it must be noted that obesity was especially associated with propionate levels.^65^

### The role of microbially derived bile acids

One group of microbially derived metabolites are secondary bile acids. A link that exists between the gut microbiome, bile acids and obesity or obesity-related diseases has been reported by many researches.^66–71^ Primary bile acids are produced in hepatocytes *via* two pathways. The classical pathway, which produces the majority of bile acids, is initiated by cytochrome P450 cholesterol 7α-hydroxylase. The alternative pathway is initiated by Cytochrome P450 27α-hydroxylase. One of the intermediates in the classical pathway, 7a-hydroxy-4-cholesten-3-one, was shown to correlate with the total plasma triglyceride concentration, indicating that hepatic bile acid synthesis is of importance in regulating the plasma triglyceride levels in obese subjects.^72^ The primary bile acids produced are cholic acid, chenodeoxycholic acid and hyocholic acid. These primary bile acids are conjugated to glycine or taurine. Post-prandial, these conjugates are secreted into bile and released to facilitate dietary fat solubilization and absorption. Hereafter, gut microbiota deconjugate the primary bile acids using bile salt hydrolases (BSHs). Many bacteria, including *Bifidobacterium* spp., *Lactobacillus* spp., *Enterococcus* spp. and *Methanobrevibacter* spp. contain these BSHs. More recently, the *Christensenellaceae* was found to contain a novel BSH.^73^ Next, these deconjugated primary bile acids are subsequently converted into secondary bile acids. This is done *via* deamination and 7α-dehydroxylation by gut microbiota. In the last stage, the bile acids are absorbed in the distal ileum, finishing the enterohepatic circulation. The secondary bile acids produced are deoxycholic acid and lithocholic acid. These bile acids are involved in regulation of energy expenditure, as well as inflammation and glucose metabolism and lipid metabolism.^74^ This indicates that these bile acids are of great interest in the pathophysiology of obesity as an alteration in the gut microbiota associated with obesity includes changes to the bile acid pool size and composition. This is because different bile acids have different affinities to various intestinal receptors^75^ such as binding to the membrane-bound Takeda G protein-coupled receptor (TGR) 5^76^ as well as with nuclear farnesoid X receptor (FXR).^77^ In mice, it has been shown that the gut microbiota promote diet-induced obesity *via* the FXR receptor.^69^ In adipose tissue, adipocyte differentiation is regulated by FXR *via* promoting peroxisome proliferator-activated receptor gamma activity, which, in turn, regulates fatty acid storage and glucose metabolism.^30^ In brown adipose tissue, energy expenditure is increased by bile acids binding to TGR5 and the subsequent production of cyclic adenosine monophosphate resulting in increased thyroid hormone activation which is involved in energy homeostasis.^31^ In macrophages, activation of TGR5 by bile acids leads to an anti-inflammatory response due to suppression of the NF-κb pathways^78^ and NLRP3-dependent inflammasome activities.^79^ Both FXR and TGR5 receptors are in similar cells such as the pancreatic β cells and enteroendocrine L cells. In pancreatic β cells, both positively regulate synthesis and glucose-induced insulin secretion. In enteroendocrine L cells, an opposing effect is observed. Activation of FXR leads to repression of GLP-1 secretion,^80^ whereas activation of TGR5 induces GLP-1 secretion.^81,82^

Several studies have correlated specific gut microbiota alterations and consequently an altered bile acid composition with obesity while taking the type of diet into account.^67–70,83–86^ A whole-grain-rich diet, compared to diet rich in refined grains, led to significantly larger amounts plasma bile acids, including taurochenodeoxycholic acid, glycocholic acid and taurolithocolic acids.^87^ This was hypothesized to activate FXR and TGR5 receptors and affect glucose homeostasis. Indeed, a vegan diet, high in dietary fibres, is associated with a high *Prevotella* abundance^84^ and was shown to enhance the FXR signalling pathway.^85^ Vegans also have significantly lower amounts of faecal bile acids compared to omnivores.^88^ When omnivores were put on a diet with increased dietary fibres, a significant reduction in faecal bile acids was observed.^86^ In mice, high-fat diet induced obesity caused increased faecal levels of deoxycholic acid.^83^ Furthermore, high-fat diet slightly increases the total bile acid pool and in particular increases deoxycholic acid and taurodeoxycholic acid levels in liver and plasma. These changes were correlated with an increased abundance of *Blautia*, *Coprococcus*, *Intestinimonas*, *Lactococcus*, *Roseburia* and *Ruminococcus.*^83^ Another mice study investigated the influence of BSHs on the FXR bile acid antagonist tauro-β-muricholic acid as FXR inhibition leads to resistance to obesity. They found that decreased *Lactobacillus* levels were correlated with decreased levels of BSH and consequently with increased levels of tauro-β-muricholic acid.^68^ Indeed, *L. johnsonii* isolated from the caecum of mice was found to express genes that produce BSHs that specifically target tauro-β-muricholic acid,^89^ providing a mechanistic link between changes in the gut microbiota and the expression of BSH genes modulating FXR. However, it remains unclear how much *Lactobacillus* contributes to the FXR antagonist concentration in comparison to other gut microbes producing similar BSHs. A human study investigating obese subjects found *Ruminococcus* from the *Lachnospiraceae* family to be positively correlated with the proportion of glycodeoxycholic acid and the ratio of secondary to primary bile acids in plasma. Besides this, *Faecalibacterium prausnitzii* was negatively correlated with isolithocholic acid levels in stool.^72^ A study investigating obese subjects observed that this group had a decreased proportion of non-12-OH bile acids. In the same study, high-fat diet obesity-resistant mice had enhanced levels of these non-12-OH bile acids. In high-fat diet obesity-prone mice, these bile acids were reduced and related to an altered gut microbiota. Here, *Clostridium scindens* was decreased.^70^ It is clear that obesity is linked with the gut microbiome *via* the bile acid pool size and composition yet there is no clear-cut link yet between single bacteria, a specific bile acid profile and the obesity phenotype. This is not surprising as the bile acid composition is clearly dependent on the microbiota composition as a whole. Therefore, more research needs to be conducted to link obesity and the bile acid profile and pool size with particular bacterial composition profiles.

### Other microbially derived metabolites related to the pathogenesis of obesity

#### Fatty acids

Besides the production of bile acids, some bacteria, including *Lactobacilli* and *Bifidobacteria*, also produce metabolites by saturation metabolism of polyunsaturated fatty acids.^90^ This results in intermediate fatty acids, such as hydroxy-, oxo-, conjugated- and partially saturated trans-fatty acids. It was shown that specific pathogen-free mice had much higher levels of hydroxy fatty acids in comparison to germ-free mice, suggesting that lipid metabolism by the gut microbiome has an influence on the fatty acid composition in the host and can therefore affect the health of the host.^91^ Furthermore, some of the fatty acids in the conjugated fatty acids group have health benefits. *In vitro* experiments on dendritic cells showed that the *cis*-9 *trans*-11 isomer of conjugated linoleic acid suppressed LPS-induced IL-12 production and enhanced the production of the anti-inflammatory cytokine IL-10.^92^ Another example is 10-hydroxy-cis-12-octadecenoic acid (HYA) as it partially regulates tumour necrosis factor receptor 2 (TNFR2), thereby facilitating an epithelial barrier recovery effect.^32^ Another study of Miyamoto *et al.* showed how HYA attenuated high-fat diet induced obesity in mice *via* GLP-1 secretion by GPR40 and GPR120. In addition, they confirmed that several species of the *Lactobacillus* genus, such as the *Lactobacillus salivarius* and *Lactobacillus gasseri*, were capable of producing HYA at similar levels, protecting the host from high-fat diet induced obesity.^93^

#### Amino acids

Production of indoles by bacteria is of importance for human health. Indoles are produced *via* catabolism of aromatic amino acids such as tyrosine, phenylalanine and tryptophan in the descending colon.^94^ Gut indole levels are thus dependent on the type of diet. A protein-rich diet promotes production of indoles.^33^ A sugar-rich diet however might lower indole synthesis as sugar overconsumption might lead to saturation in the small intestine which could lead to more remaining sugar entering the large intestine. As carbohydrate fermentation is preferred over proteolytic activity, thereby inhibiting tryptophanase activity leading to a lower rate of indole synthesis.^95^ Indole influences host metabolism *via* modulation of GLP-1 secretion by L-cells,^33^ indicative of playing a role in metabolic diseases such as T2D. Indole-3-propionic acid is an indole, produced by *Clostridium sporogenes*,^96^ which is positively correlated with dietary fibre intake.^97^ Indeed, one study found an association between higher plasma levels of indole-3-propionic acid and reduced risk for the development of T2D.^97^ Another study found reduced levels of indole-3-propionic acid in obese subjects with T2D when compared to lean controls.^98^ Indole-3-propionic acid was shown to regulate inflammation by binding to the pregnane X receptor and subsequently downregulating TNF-α.^34^ Furthermore, indole-3-propionic acid has been shown to reduce gut permeability in diet-induced obese mice.^98^ Indole-3-carbinol has also been shown to have anti-obesogenic activity in mice.^99^

In the gut, tryptophan can be used as a substrate by the gut microbiota to produce indoles, but can also be metabolized by the host.^100^ During low-grade intestinal inflammation, a chronic symptom of obesity, 2,3-dioxygenase activity in macrophages is increased leading to higher production levels of kynurenine, diverting production away from microbially derived indoles. Mice on a high-fat diet show increased indoleamine 2,3-dioxygenase activity compared to mice on a normal chow diet. However, an improvement in insulin tolerance was observed in mice in which this enzyme was knocked-down, compared to wild-type mice on a high-fat diet.^100^ This improvement in insulin tolerance is mediated *via* the aryl hydrocarbon receptor as its activation reduces insulin resistance and inflammation in epidydimal white adipose tissue.^100^ Aryl hydrocarbon receptor activation also causes a production of IL-22 and inhibition of inflammation in the GI-tract.^35^ Microbially derived indoles such as indole-3-acetic acid activate the aryl hydrocarbon receptor but kynurenine inhibits its activation. Microbially derived indole-3-acetic acid furthermore limits fatty acid accumulation and production of inflammatory markers in macrophages.^101^ Studies in humans have shown that plasma level and faeces level of kynurenine are associated with obesity as they are higher in obese subjects than in controls.^100^ Taken together with the changes induced by a high-fat diet on the gut microbiota,^102^ this provides yet another example how diet influences the gut microbiome and how this affects inflammation and obesity in the human host.

Besides indoles, other amino acids can also influence the host. An example is glutamate, which was shown to be potentially harmful according to a genome-wide association analysis of a cohort comparing obese and lean subjects.^103^ By conducting pathway analysis, the glutamine/glutamate transport system was shown to be highly enriched in obese individuals. Correlation analysis showed an inverse correlation with species from *Bacteroides*, including *B. thetaiotaomicron*. Indeed, obese individuals had a decreased abundance of this bacterium compared to lean subjects.^103^ Investigating the role of *B. thetaiotaomicron* in mice on a high-fat diet indicated that the expression of genes encoding for proteins involved in lipogenesis was lower and that the expression of genes encoding for proteins involved in fatty acid oxidation and lipolysis was higher. Also, the expression of markers involved in inflammation was lowered.^103^ One side note the authors made in regard to finding the *B. thetaiotaomicron* related to obesity, was that the effect might be due to interaction with certain additional species, such as the *B. uniformis*,^103^ which is known to partially restore the effect of high-fat diet induced obesity.^104^

## Bacteroidetes to Firmicutes ratio

A controversial topic supposedly separating healthy subjects from their obese counterparts is the Bacteroidetes to Firmicutes ratio. This ratio was first mentioned in the study of Ley *et al.*, investigating the differences between the caecal microbiota of genetically predisposed obese mice and their lean wild-type siblings receiving the same polysaccharide-rich diet. In the obese mice, Bacteroidetes numbers were reduced while the relative abundance of Firmicutes was higher.^105^ A year later, similar results were found when comparing obese and lean humans.^106^ However, controversial results were observed by the same group when comparing lean human and obese human twins. Here, a significant decrease in Bacteroidetes was observed, however, not in relation to Firmicutes.^107^ On top of this, re-analysing both datasets of the previous mentioned articles and other publicly available data using similar pipelines and region of the 16s rRNA gene also led to contradictory results in relation to Bacteroidetes to Firmicutes ratio.^108–110^ These contradictory gut microbiota results on the phylum level are not surprising, given the multitude of orders, families, genera and species represented by both of these phyla that inhabit the human intestinal tract. Comparing phylum levels with one another, for example a Bacteroidetes to Firmicutes ratio, is really like comparing blue whales to starfish (both Animalia). The Firmicutes phylum on the other hand is so broad that saying something is a Firmicute literally tells one nothing about the function of that bacterium. This is in contrast to phyla such as the Verrucomicrobia or the Euryarchaeota that include only a few relevant species from a human intestinal tract point of view. In addition, taxonomically different bacteria within these phyla have vastly different attributes. The most important example within the Bacteroidetes phylum are the *Prevotella* and *Bacteroides* genera which tend to be mutually exclusive.^111,112^ Conflicting results are to be expected when pooling bacteria together per phylum when comparing multiple studies. Therefore, the use of the Bacteroidetes to Firmicutes ratio is discouraged.

## *Prevotella* to *Bacteroides* ratio

After the introduction of the enterotypes,^113^ a new ratio was coined by Roager *et al.* As alluded to above, a more suitable distinction was made within the Bacteroidetes phylum, namely the *Prevotella* and *Bacteroides* ratio.^114^ This was initially due to an article of Koeth *et al.* in which it was observed that individuals with the *Prevotella* enterotype had higher plasma concentrations of trimethylamine-N-oxide when consuming l-carnitine (present in red meat) compared to the *Bacteroides* enterotype.^115^ A *Prevotella*-dominant gut microbiome tends to be associated with vegetarianism or with a non-industrialized dietary fibre (plant)-rich diet. Examples of such *Prevotella*-rich microbiomes can be found in several studies performed hunter-gatherers or rural populations in Africa,^116–118^ South America^119^ or South-east Asia.^120^ The *Bacteroides* enterotype is more associated with Western industrialized populations and is especially dominant in the United States.^116,117,119,120^ A shift away from *Prevotella* towards a more *Bacteroides* dominant gut microbiome as a result of diet and environment is nicely exemplified by the study by Vangay *et al.*,^120^ where people from rural Thailand migrated to the United States. Unsurprisingly, this shift was also accompanied by an increase in weight. In regard to weight loss regiments, this ratio is important as subjects with a higher *Prevotella* to *Bacteroides* ratio have been shown to be more prone to weight loss when given a diet high in dietary fibre/whole grain.^112,120^ On the contrary, subjects with larger amount of *Bacteroides* were found to lose more weight loss when given capsaicin,^121^ emphasizing the need for personalized nutrition.

## Rationale for taking a guild-based approach to obesity

Most research done on the relation between obesity and the gut microbiota usually links up individual taxonomic group to (a) pathophysiological pathway(s) to establish a connection with obesity. Bacterial species however do not exist in a vacuum and their growth rate and even which metabolic activities they can perform depend upon external environmental factors. These external factors include things such as pH,^122^ bile acids^123^ and substrate availability.^124^ All of these are, in turn, dependent on the microbiome composition itself; this means that the function of one bacterial species depends on or is influenced by all other bacterial species surrounding it. Even more directly to the issue at hand, various bacterial species depend upon other bacterial species to provide them with intermediate substrates (waste products of other bacteria) and are, in turn, dependent on other bacteria that will consume their own waste products (fermentation products) in order for their biochemical conversions from which they derive energy to be energetically favourable.^53^ Lacking the right microbial partners bacteria/archaea/fungi may not even be able to utilize particular metabolic pathways (for very long), making it necessary to perform other metabolic tricks to produce adenosine triphosphate (ATP). And this is only a tip of the iceberg in regard to the manner of ways in which bacteria of various species interact. Competition for limited resources is an important issue, quorum sensing that can be used by bacteria to sense the presence and quantity of other bacteria to facilitate communication between mutualistic bacterial species or to make the life of competing microorganisms difficult are other factors.^125^ By simply examining a single bacterium without any context (its surrounding microbiome), it is to be expected that when comparing different studies/cohorts that opposing results and interpretations can easily be obtained.

Adding to the complexity is that different taxonomical levels (phylum/family/genus/species) are often being used to attribute particular characteristics and associations while the function of species even within the same genus, or even different strains of bacteria currently considered to be of the same species, can differ wildly (as alluded in the ‘Bacteroidetes to Firmicutes ratio’). Dimension reduction strategies that aim to limit the number of taxonomic groups by looking at a higher taxonomic level should thus be usually preferably limited to a genus-like level. Notwithstanding, analysing datasets at the species or strain level might also obfuscate relevant patterns if all species/strains from a particular genus are all more or less doing the same thing. Lastly, complications can arise if one uses reference database-dependent methods to quantify microorganism if those microorganisms are not well represented in the reference database resulting in a large fraction of ‘unknown’ reads. This is an issue for analysing bacterial profiles but for gene centric-based analyses, besides the issues of high dimensionality and sparsity, the limitation of available reference databases will also cause reads not to map to particular gene catalogues, leading to the omission of possibly meaningful data.^126^ Further complicating matters, different strains of the same species might or might not have particular functions attributed to them, as is observed in carbohydrate-active enzymes.^127^ Conflicting patterns may furthermore occur for highly similar genes if they are present in multiple bacteria.^126^

From a more birds-eye point of view, an increasing number of authors have concluded within the last few decades that a beneficial effect in relation to obesity should be attributed to multiple players within the gut microbiota working together,^118,126,128^ whereas the disturbance of such associations can be seen as a form of dysbiosis.^129^ As the aforementioned drawbacks of the analysis of individual taxonomic groups make it difficult to find biologically meaningful patterns specific to health outcomes, two different terms were coined to collapse individual microbiome members into groups. Zhang *et al.* applied the term ‘guild’, which was already known in macro-ecology.^130^ It includes ‘a group of species that exploit the same class of environmental recourses in a similar way’, which later became synonymous with ‘functional groups’. In the elegant opinion article of Wu, a framework is given to disentangle the relationship between the gut microbiome and human health in a more ecological meaningful fashion by constructing co-abundance groups based on microorganisms covariation of abundance.^126^ This will overcome a wide variety of drawbacks that are currently of issue for taxon-based analysis and gene-centric analysis. Another term, which can fall under the umbrella term of ‘guilds’, is called ‘trophic network’, which was, for example, utilized in an article of de Goffau *et al.*^118^ Here, micro-organisms working together in a syntrophic relationship are of interest. In this article, the gut microbiota of children aged 7- to 37-month old living in rural, The Gambia, was investigated to study the development of the gut microbiome over time. A trophic network is defined as a microbial population forming a food web of metabolically interdependent organisms which builds up steadily over time in a correlated fashion.^118^ A straightforward example which is part of a trophic network is observed in the production of butyrate by means of cross-feeding, as mentioned in the section ‘The role of SCFAs’. More meaningful interpretations of gut ecology in relation to health and obesity may thus be achieved by looking at guilds of bacteria or specific trophic networks. This is further depicted in the article of Wu *et al.* Here, the gut microbiome of patients (both obese and non-obese) with polycystic ovary syndrome were investigated and compared to controls not having the disease (both obese and non-obese). Performing taxon-based analysis led to a positive correlation between *Bacteroides* and the disease. However, when performing guild-based analysis, *Bacteroides* operational taxonomic units (OTUs) were further divided in different guilds, where some guilds show a positive correlation to the disease and other guilds show negative correlations with the disease. This example highlights how discordant results between studies could arise in regard to *Bacteroides* when doing taxon-based analysis as members of the same taxon can have opposite relationships with the disease phenotype.^126^ Furthermore, clustering hundreds of taxonomic groups into a limited number of guilds or trophic networks will help reduce dimensionality, giving the possibility to apply classical statistics limiting the problems associated with correcting for multiple testing. Though guild-based approach seems a promising approach, with added value observed in understanding weight regulation of obese children,^126^ the relevance on obesity itself remains to be elucidated.

## Trophic networks relate to microbial diversity and health

A common observation when distinguishing obese subjects from their healthy counterparts is their average lower α-diversity.^103,107,110,120,131^ The same is observed in many other diseases, such as Crohn’s disease, irritable bowel syndrome and colorectal cancer. Thus, a loss of microbial diversity is commonly associated with various diseased states. It can be said that, post-weaning, a lower gut α-diversity, is a general feature associated with various human conditions. In adult humans, a higher abundance of bacteria such as the *Akkermansia muciniphila* and *F. prausnitzii* is typically associated with a higher α-diversity. Indeed, the abundance of *A. muciniphila* is negatively associated with BMI, inflammatory markers, lipid synthesis and total adipose tissue weight.^55,132–135^ α-diversity is shaped by a combination of dispersal, local diversification, environmental selection and ecological drift. Diversity by itself is not just an indicator of health as a highly diverse mix of pathogens will certainly not induce intestinal bliss. Instead, a higher α-diversity should be seen as the presence of (several partially overlapping) well-developed and extended microbial trophic networks that together lead to an improved fermentative capacity. *Bacteroides*-rich microbiomes tend to have lower α-diversity values, simpler less extensive trophic networks and are more prone to descend, mediated for example by diet or other factors, into low α-diversity compositions which should be considered truly dysbiotic. Such low α-diversity compositions are typically enriched in species such as *Enterobacteriaceae*, *Fusobacterium, Streptococcus, Ruminococcus gnavus* and/or various *Bacteroides* species. Complex intertwined co-dependencies of species in such compositions are typically taking a backseat (breakdown of trophic networks). Such dysbiotic compositions are when described in terms of enterotypes best likened to the *Bacteroides* 2 enterotype and ultimately are a risk factor among others for obesity^129^ and T2D.^136^ Not surprisingly, in an article of Vieira-Silva *et al.*,^129^ the *Bacteroides* 2 enterotype is also linked to high C-reactive protein production by the liver, indicative of inflammation.

A telling example of the effects of a total destruction of trophic networks and consequently extremely reduced α-diversity, reduced gene richness and gut fermentative capabilities is the study by Wijeyesekera A *et al.*^51^ where they investigated the gut microbiome profile, the faecal SCFA profile and the bile acid profile of (antibiotic treated) critically ill children. The ratio of primary to secondary bile acids was higher in these children as a result of a lack of metabolic and fermentative capability, but also the production of SCFAs (end products such as acetate, butyrate and propionate) was critically low while the levels of intermediate products of carbohydrate fermentation such as lactate and succinate were increased, as compared to the healthy control children. The latter finding, together with remaining unfermented sugar fractions and higher levels of untouched proteins and much looser stool (diarrhoea even), highlights how the remaining fermentation in the gut was still somewhere in the saccharolytic phase. Various bacterial species still somewhat abundant within these children were typically representatives of the *Bacteroides 2* enterotype as described above.

One particular trophic network that is consistently associated with a high α-diversity and health, specifically leanness, includes the *Christensenellaceae* family.^131,137^ Importantly, a review article by Waters and Ley summarizes numerous articles that describe finding higher *Christensenellaceae* levels in healthy subjects with a normal BMI (between 18.5 and 24.9 kg/m^2^) compared to obese subjects.^138^ The association between *Christensenellaceae* and host BMI is considered to be one of the most robust associations. Transplantation of *Christensenellaceae minuta* enriched faeces from a human donor in germ-free mice led to reduced adiposity.^139^ *Christensenellaceae* are commonly abundant in those people who are said to be of the *Ruminococcaceae* or Firmicutes-enriched enterotype. As mentioned, the *Christensenellaceae* family should not be seen as a distinct stand-alone entity as it consistently forms a trophic network with other bacteria and archaea which are similarly associated with low BMI. The association of *Christensenellaceae* with *Methanobrevibacter smithii*, an Archaea, is probably the best described and understood part of this trophic network.^139,140^ *Methanobrevibacter smithii* produces methane from the hydrogen that is produced by the *C. minuta.*^140^ If there is a causal relationship between this trophic network and low BMI, it is however still rather uncertain. Some hypothesize that the supposed healthy lean effect of *Christensenellaceae* is amplified by *Methanobrevibacter* as the consumption of hydrogen by *Methanobrevibacter* makes production of acetate by *Christensenellaceae* favourable over butyrate.^140^ Limiting butyrate production of *Christensenellaceae* is in this hypothesis thereby thought to limit the availability of energy for the human host colonocytes. While acetate can also be taken up and utilized as an energy source elsewhere in the human body, it is not as energy rich from an aerobic respiratory point of view. Others would however argue against this hypothesis as they find butyrate production to be beneficial in various ways, including protection against obesity and obesity-related diseases^141,142^ or say that it is not always clear and might depend on the larger context.^143^ In addition to *M. smithii* being part of this trophic network, a study comparing lean to obese elderly in Italy found a correlation between *Christensenellaceae*, *Rikenellaceae and Porphyromonadaceae.*^131^ In a cohort from Japan, investigating faecal samples from healthy adults in different regions, *Christensenellaceae* was also negatively associated with BMI together with various other bacteria, including the *Dehalobacteriaceae*, *Desulfovibrionaceae*, *Mogibacteriaceae*, *Odoribacteraceae*, *Oxalobacteraceae*, *Peptococcaceae*, *Rikenellaceae*, *Ruminococcaceae*, *Synergistaceae*, *Verrucomicrobiaceae* and *Victivallaceae.*^144^ Given the strong link between α-diversity, leanness and the *Christensenellaceae* trophic network of bacteria, there is a strong incentive to investigate this association mechanistically. It should also be noted that the importance of this trophic network in regard to SCFA production has yet to be determined. On one hand, *C. minuta* only produces limited amounts of butyrate (0.3 mM) and acetate (3.6 mM) *in vitro*.^145^ On the other hand, while *Christensenellaceae* and *Methanobrevibacter* might together only constitute a small proportion of the total microbiota, the trophic network of which they represent core indicator species is by no means a small player in various ethnicities. This trophic network, in which various species are very strongly correlated with one another, is of enterotype defining potential.^131,144^

Another trophic network, that is typically underrepresented in people living in industrialized countries, is the *Prevotella stercorea* trophic network, which can be seen as an important factor within *Prevotella* enterotype compositions. The build-up of this trophic network was first extensively described by looking at the developing gut microbiome of children from The Gambia. The *P. stercorea* forms a large trophic network with, among others, *Succinivibrio dextinosolvens* and *Paraprevotella xylaniphila*, and is similarly associated with a high α-diversity. Interestingly, *Bacteroides* and species associated with (the) *Bacteroides* enterotype(s) are not observed to be highly abundant in The Gambia, in contrast to industrialized countries in which obesity is rising at the highest rate.^120^ The *Prevotella* enterotype itself is also associated with a lower BMI^120,146^ and an inverse correlation between low-density lipoprotein cholesterol (LDL-C) and the *Prevotella* enterotype has been observed, indicating that *Prevotella* enterotype is associated with health in non-industrialized countries.^146^ A lot of discussion exists about the use and or even existence of enterotypes. While we do not agree with the existence of discrete enterotypes we rather see it as a continuum, with various more preferred/stable states. Enterotypes nonetheless remain a very useful concept for studying and understanding the human microbial community landscape.^147^

The *Prevotella* enterotype is furthermore a perfect example to showcase the difference between co-occurrence and a trophic network. In population-wide studies, for example using data from the multi-ethnic HELIUS cohort study, people who are defined as having the *Prevotella* enterotype typically have very high *Prevotella copri* levels and high levels of species associated with the *P. stercorea* trophic network.^148^ Typically, *P. copri* and species from the *P. stercorea* trophic network cluster together when visualized in a hierarchically clustered heatmap. This co-occurrence is however mainly the result of the strong antagonism between fecal *Prevotella* (including *P. copri*, *P. stercorea* and other many other *Prevotella* sp.) and *Bacteroides*/*Phocaeicola.*^112^ Both *P. copri* and the *P. stercorea* trophic network do well in the same environment (*Bacteroides*-poor), yet the high abundance of *P. copri* develops completely independently from the *P. stercorea* trophic network, as can be seen by tracking the gut microbiota maturation of children during the first 3 years of life living in an environment where everybody develops a *Prevotella*-rich gut microbiome.^118^ *P. copri* becomes and stays dominant after 12 months while abundances of species associated with the *P. stercorea* trophic network increase slowly and in a co-dependant fashion during the first 30 months of life before reaching a stable level. Exchange of various metabolites is assumed within the *P. stercorea* trophic network but deserves further investigation, especially in relation to the increased capacity of the *Prevotella* enterotype in regard to SCFA production.^117,149^

## Next steps on investigating the role of the microbiome in relation to obesity

### FMT

Although association studies using large cohorts are essential in attempting to disentangle the extreme complexity of the gut microbiome in relation to obesity, several other avenues of investigation also have potential. One such avenue is the use of FMT. FMT, also referred to as ‘human intestinal microbiota transfer’, ‘faeces transplantation’ and ‘faecal bacteriotherapy’, is the transfer of faeces from a lean donor to a recipient.^150^ The first documented use of FMT as a therapy dates back to the Dong-Jin dynasty in the 4th century by Ge Hong in which it was applied as treatment of severe food poisoning and diarrhoea.^151^ FMT has been shown to be a more effective treatment for recurrent *Clostridiodes difficile* infection (CDI) than antibiotics.^152^ Unlike obesity however, from a pathological point-of-view, CDI is a relatively straightforward disease in which the causality of the gut microbiota is clear.^150^ In a FMT trial on obese subjects with insulin resistance by Vrieze *et al.*^153^ subjects either received their own faeces (autologous) or lean-donor faeces (allogenic). A short-term (6 weeks after FMT) beneficial effect on insulin sensitivity (based on the rate of glucose disappearance) was observed in subjects receiving lean-donor FMT. Further investigation by Kootte *et al.*,^154^ showed that the baseline gut microbiota predisposes FMT success (defined by at least a 10% increase rate of glucose disappearance). Here, FMT success occurred more frequently in subjects with decreased α-diversity when an allogenic FMT was received.^154^ A similar trend was observed in another pilot study of Yu *et al.*^155^ These subjects with a lower α-diversity are likely to have a Bacteroides 2-type enterotype. As mentioned, this includes a lower microbiome gene richness and fermentatively a less capable composition compared to, for example, the *Ruminococcaceae* enterotype.^129^ Simply put, in those subjects with a low α-diversity, there is more room for improvement than in subjects whose gut microbiome composition has not yet deteriorated as much. In addition, a study by Podlesny *et al.*, that included several FMT cohorts investigating different diseases, showed both ecological variables, such as low α-diversity, play a role in engraftment success together with clinical variables, such as antibiotics treatment and lavage. They furthermore showed that increasing the α-diversity by pooling donor samples was predicted not to increase donor strain engraftment, indicating that pooling donor samples is not functionally equivalent to a single high α-diversity donor sample.^156^ Subsequent analyses on the cohort by Kootte *et al.* revealed that the *P. copri* had a beneficial effect in subjects receiving an allogenic FMT. *P. copri* was further negatively correlated to BMI, C-reactive protein and fasting insulin levels.^157^ Furthermore, changes in the gut microbiota could be linked to particular plasma metabolite levels and changes in DNA methylation in plasma blood mononuclear cells (PBMCs),^157^ providing additional clues on the mechanisms with which the gut microbiome affects obesity-associated disorders.

### Bioinformatic tools to verify strain engraftment success

**Figure 2.**
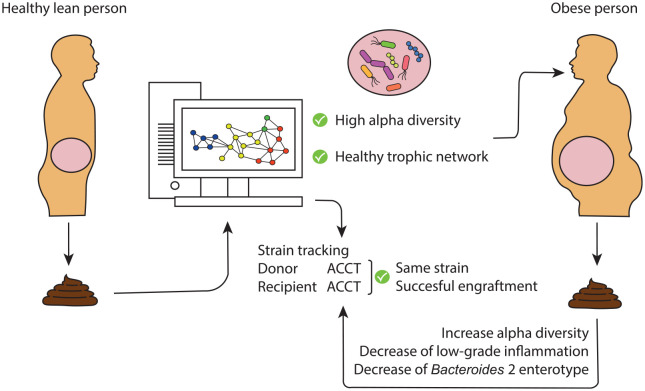
Schematic overview of a promising approach to alleviate obesity and associated diseases of burden. The microbial composition of the faeces of healthy lean donors is analysed to select for donors with a high α-diversity (among others), which can be seen as a marker for the presence of complex health-associated trophic networks. If suitable, faeces of a high α-diversity donor is then transferred to an obese recipient, potentially alleviating low-grade inflammation. Donor strain verification with specific SNPs (ACCT in the figure) on specific positions in the genome of the gut microbiota are traced after FMT in the feces of the recipient using strain tracking.

Several tools have recently been developed to help disentangle the relationship between the gut microbiome and obesity in the context of FMT. To verify that strains from a lean donor are engrafted in a recipient, strain tracking analyses need to be conducted (Figure 2). A benchmark article published last year compared seven different bioinformatic tools for strain tracking on the HMP dataset. Here, it was observed that probabilistic tools perform best on short-read metagenomic sequencing data.^158^ This field of technology is however still developing rapidly with two new strain tracking tools recently developed. One of these tools is tracking strains based on single nucleotide variants in the species-specific marker genes^159^ and the other is an improved and further build tool which was previously published.^160^ Applying the strain tracking methodology, Li *et al.*, who investigated strain engraftment in recipients upon receiving FMT, observed that donor- and recipient-specific strains can coexist.^161^ This method of strain tracking was also applied by Wilson *et al.* Here, they showed that FMT capsules in obese subjects led to changes in the microbial community composition causing subjects shift from one enterotype to another. This subsequently changed the metabolic potential of the community. The microbiome shift towards a donor was positively correlated with α-diversity.^162^ Furthermore, changes in the gut microbiota composition persisted 26 weeks after treatment. Moreover, this study combines faeces of multiple donors and showed that some donors have highly effective microbiomes for engraftment,^162^ which implicates the important role of the composition of the donor faeces and the transfer of entire trophic networks instead of the addition of single taxonomic groups.

### Machine learning to gain insights into the pathogenesis in obesity

Multivariable predictive machine learning models represent a powerful statistical method to understand the biology behind obesity. Given the high dimensionality of -omics data, univariate statistical significance only leads to single markers that, after multiple testing correction, may not be significant anymore. Multivariate machine learning models provide several advantages over classical statistics. These include the ability to take nonlinear relationships between biomarkers into account and the formation of a panel of reliable biomarkers, rather than single biomarkers as would be obtained using classical statistics. Stratified shuffle split, cross-validation and rigorous stability selection procedures^163^ are applied in these models to prevent overfitting to acquire a panel of reliable biomarkers. Permutation of the output variable yields a validation on the reliability of the area under the curve found.^164^ Permutation analysis also circumvents the need for multiple test correction, applied in classical statistics. A study distinguishing subjects with T2D from their matched controls based on the microbiome profile used such a nonlinear machine learning model with rigorous stability selection.^165^

Multi-omics machine learning models help understand the complex multifactorial aetiology of obesity. Usually, multiple -omics profiles, medical records and other unstructured data sources are present. The core mechanism in a health state or disease state is a cohesive entirety of multiple modalities. An example in which we looked at multiple -omics modalities is during the follow-up analysis of the FMT study of Kootte *et al.*^154^ Support vector machine models were deployed on different -omics panels, which yielded highly discriminatory areas under the curve and important biomarkers on each -omics modality. Correlations of the most discriminative biomarkers over all -omics modalities suggest a specific interconnection of gut microbiota, plasma metabolites and DNA methylation loci in PBMCs.^157^

## Conclusions

Obesity and the gut microbiome are intertwined in a myriad of ways. The type of diet and its quantity logically affect the availability of energy and hence obesity but also strongly affect the gut microbiome which, in turn, can amplify the obesogenic properties of a diet or on the other hand provide various protective benefits. The immune system, for example, recognizes bacterial LPS *via* PRRs causing adipocytes to produce proinflammatory cytokines while the recognition of peptidoglycan *via* the NOD2 PRR attenuates inflammation. Many microbially derived metabolites, including SCFAs, bile acids, indoles and other amino acids, are similarly critical for health. An excess or lack of these, or more specifically, an altered overall composition in any of these modalities, may be obesogenic. Commonly, microbial taxa are individually associated with pathogenesis when comparing obese subject with lean controls. This can however lead to contradicting findings as particular microorganisms within different microbial compositions can have different functions. One should thus instead look at the overall microbial composition in regard to its effect on all of the other modalities. In addition, one should strive to at least utilize a sufficient taxonomic resolution when analysing a composition. A phylum-level analysis, for example comparing the Bacteroidetes to Firmicutes ratio, has led to many discordant findings which is not surprising as these phyla comprise many different bacterial families which, in turn, harbour numerous different species that are functionally very different and/or are even competing with each other. Within the Bacteroidetes phylum example, the *Prevotella* to *Bacteroides* ratio is more biologically relevant. The *Prevotella* enterotype, for example, tends to be associated with a non-industrialized dietary fibre-rich diet. The *Bacteroides* enterotype is more associated with the Western industrialized populations. A shift from the *Prevotella* to the *Bacteroides* enterotype is typically associated with an increase in weight. However, when comparing diseased individuals to healthy individuals, different *Bacteroides* OTUs have been both positively and negatively correlated with disease again highlighting the limitations of a taxon-based approach.

A better approach to disentangle the complex relationship between bacteria and obesity is a guild-based or trophic network-based approach. Opposed to gene-centric or taxon-based approaches, grouping multiple (phylogenetically) different bacteria in the same group based on their interdependency can establish a stronger connection to obesity. A trophic network of various species that is centred around *Christensenellaceae* is, for example, associated with increased α-diversity and healthy BMI values. The *Prevotella* enterotype, and concomitantly the many species associated with *Prevotella*, is also linked with lower BMI values and is inversely correlated to LDL-C. This is possibly because the main competitor enterotype of the Prevotella enterotype is centred around *Bacteroides*, which is associated with a more Western diet. Within the *Bacteroides*-dominated gut microbiota composition types, the dysbiotic ‘Bacteroides 2-enterotype’ is especially prevalent among obese subjects and is strongly positively correlated with BMI. Furthermore, this latter enterotype is linked to lower gene richness, resulting in a fermentatively less capable composition and higher levels of C-reactive protein.

There are several avenues to take the complex interaction between the gut microbiome and the pathogenesis of obesity into account. One such avenue, first used to treat patients with recurrent *C. difficile* infection, is FMT. FMT was further investigated in subjects with MetSyn and showed that allogenic FMT could improve insulin sensitivity. The success of FMT might lie in the fact that it can introduce entire trophic networks, using the right donor, instead of just adding a single supposedly beneficial bacterium. Furthermore, different bioinformatic tools and machine learning can further help to understand the complex pathogenesis of obesity. Strain tracking can be applied to verify that bacteria engraft from a lean donor in an obese subject receiving FMT. In addition, different -omics modalities and combining these modalities in machine learning serve as a powerful tool that can take nonlinear relationships into account to find reliable combinations of biomarkers in the pathogenesis of the gut microbiome in obesity.

## Declarations

**Ethics approval and consent to participate:** Not applicable.

**Consent for publication:** Not applicable.

**Availability of data and materials:** Not applicable.
